# Environmental Roots of the Late Bronze Age Crisis

**DOI:** 10.1371/journal.pone.0071004

**Published:** 2013-08-14

**Authors:** David Kaniewski, Elise Van Campo, Joël Guiot, Sabine Le Burel, Thierry Otto, Cecile Baeteman

**Affiliations:** 1 Université Paul Sabatier-Toulouse 3, EcoLab (Laboratoire d’Ecologie Fonctionnelle et Environnement), Toulouse, France; 2 CNRS, EcoLab (Laboratoire d’Ecologie Fonctionnelle et Environnement), Toulouse, France; 3 Institut Universitaire de France, Paris, France; 4 CEREGE, Aix-Marseille Université CNRS UMR 7330, Europôle de l’Arbois, Aix-en-Provence, France; 5 Geological Survey of Belgium, Division Earth and History of Life, Royal Belgian Institute of Natural Sciences, Brussels, Belgium; University of Oxford, United Kingdom

## Abstract

The Late Bronze Age world of the Eastern Mediterranean, a rich linkage of Aegean, Egyptian, Syro-Palestinian, and Hittite civilizations, collapsed famously 3200 years ago and has remained one of the mysteries of the ancient world since the event’s retrieval began in the late 19^th^ century AD/CE. Iconic Egyptian bas-reliefs and graphic hieroglyphic and cuneiform texts portray the proximate cause of the collapse as the invasions of the “Peoples-of-the-Sea” at the Nile Delta, the Turkish coast, and down into the heartlands of Syria and Palestine where armies clashed, famine-ravaged cities abandoned, and countrysides depopulated. Here we report palaeoclimate data from Cyprus for the Late Bronze Age crisis, alongside a radiocarbon-based chronology integrating both archaeological and palaeoclimate proxies, which reveal the effects of abrupt climate change-driven famine and causal linkage with the Sea People invasions in Cyprus and Syria. The statistical analysis of proximate and ultimate features of the sequential collapse reveals the relationships of climate-driven famine, sea-borne-invasion, region-wide warfare, and politico-economic collapse, in whose wake new societies and new ideologies were created.

## Introduction

Compared to the Last Glacial period, the amplitude of Holocene climate fluctuations is less pronounced. Nevertheless, climate fluctuations on multi-centennial to millennial time-scales and rapid shifts occurred during the Holocene [1]–[4], and significantly impacted human societies [5]–[10]. New light has recently been shed by both paleoclimate [11]–[16] and archaeological communities 17–19 on the so-called 3.2 ka event, also termed Late Bronze Age (LBA) collapse or crisis [20]–[21] This event was associated with a major cultural disruption at *ca.* 1200 BC, with population migrations and wars. From this crisis arose new societies and new ideologies [22].

Before the LBA crisis (ca. 1200 BC), the Eastern Mediterranean hosted some of the world’s most advanced civilizations. In the Aegean, the Mycenaean culture was flourishing with powerful urban centres such as Mycenae and Tiryns in Argolis, Pylos in Messenia, Athens in Attica, Thebes and Orchomenus in Boeotia, Iolkos in Thessaly, and Knossos in Crete [23]. The Hittites had carved out a vast empire encompassing a large part of Anatolia, the north-western region of Syria, and extending eastward into Upper Mesopotamia [24]. In the Levant, the Canaanite coastal cities were prospering through trade from Egypt to Mesopotamia, and Canaan was the sphere of interest of the Egyptian and Hittite empires [25]. In Egypt, the New Kingdom was at its height during the prosperous reign of Seti I (first regnal year: 1307–1296 cal yr BC; historical date 1295–1279 BC) and Ramses II (first regnal year: 1292–1281 cal yr BC; historical date 1279–1213 BC) [26]–[27]. However, around 1200 BC, at the end of the LBA, the Eastern Mediterranean civilization declined or collapsed 20,28–33. Several not mutually exclusive causes were put forward to explain the situation of decline, including natural disasters (tsunami, earthquake), technological innovations, internal collapses, and anthropological or sociological theories dealing with states of inequality and the resulting political struggle between urban centre and periphery [20], [21], [24], [30], [34]–[39]. Written sources from Ugarit (Syria) and Medinet Habou (Egypt) [32], [34], [40]–[42] revealed that the final catalyst that put the fall of cities and states in motion was due to vast movements of seafaring and inland tribes, the Sea Peoples [43]–[44]. The last written correspondences between Levantine, Hittite, and Egyptian kings indicate that these nomadic raiders were of great concern for the coastal towns as well as great empires and vassal kingdoms [34].

Despite the abundant literature devoted to the Sea peoples, we still do not know exactly who they were, where they came from, why they attacked, and, finally, where they disappeared to after their raids. Some scholars are even uncertain whether the Sea People’s existence was a cause or an effect of the decline of the LBA. Recent studies [12], [22] have suggested that the Sea People raids were the final step in a long and complex spiral of decline in the Eastern Mediterranean world, and that this unstable period was, at least partly, related to environmental causes operating over sizable areas. A climate shift, centred on the 13^th^–9^th^ centuries BC, could not be without consequences on Eastern Mediterranean and West Asian environments where dry farming agro-production, pastoral nomadism and fishing were the primary or secondary subsistence systems. Reduced precipitation probably affected the outlying nomad habitats, and led rain-fed cereal agriculturalists to habitat-tracking when agro-innovations are not available [8], [45]–[46]. According to this theory, the enigmatic Sea Peoples were not a bunch of pirates merely in pursuit of the richness of the Near East, but they rather constituted a set of ethnic entities fleeing inhospitable regions to conquer new lands, already undermined by the widespread drought [22].

However, the debate about the role of climate in shaping ancient societies has emerged because a direct causation fails to be proven [47], specifically when human-climate linkages are proposed at a wide regional scale. An unequivocal spatial and temporal fit between both, confirmed by local written evidences, may offer a valuable case for emphasizing an ecological-climate influence on past societal shifts.

**Figure 1:**
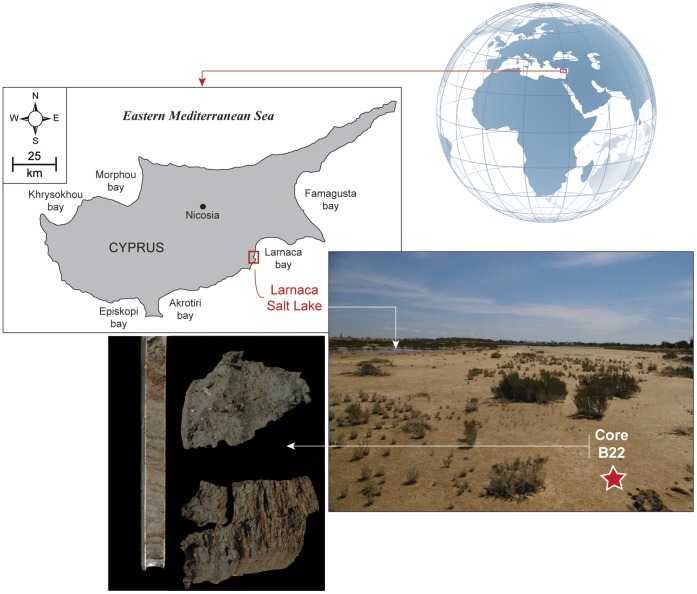
Map of Cyprus with an overview of the Larnaca Salt Lake (Hala Sultan Tekke) in the Larnaca Bay. Core B_22_ is indicated by a red star in the modern salty area. High concentrations of *Posidonia oceanica* fibers are highlighted in the corer, and in lower samples.

Here, we focus on Cyprus, an island located at the heart of the ancient civilizations and trade routes of the Eastern Mediterranean during the LBA [48]. A numerical-derived climatic proxy based on a pollen record from Hala Sultan Tekke, Larnaca Salt Lake, details the environmental context along the southeastern Cypriot coast during the LBA crisis (Fig. 1). Data from Hala Sultan Tekke were correlated with the climate reconstruction from Gibala-Tell Tweini, a thriving trade center located on the coast of the Ugarit kingdom in Syria, which provided the only known radiocarbon (^14^C) date for the LBA crisis and the Sea People raid. Cyprus and Syria had a long and rich story of commercial relationships before the Sea People event. They now meet through a common climate story to decipher the environmental roots of the LBA crisis.

## Materials and Methods

### Core Lithology and Chronology

The continuous core B_22_ (34°52′51.15″N, 33°36′43.68″E) was sampled in the Larnaca Salt Lake, near Hala Sultan Tekke, in southeastern Cyprus. No specific permissions were required for this location and this type of activity because the lake belongs to the European Union and is free of access for European citizens. The field studies did not involve endangered or protected species. The layers identified in the cores were radiocarbon dated by accelerator mass spectrometry (AMS) ^14^C on short-lived samples (Table 1). Conventional ^14^C ages were calibrated using the program Calib Rev 6.0.1 with IntCal09 [49]. The base of the core B_22_ is radiocarbon dated at 3290±30 ^14^C yr BP (820 cm depth, intercept: 3540 cal yr BP; 2 sigma (σ) calibration: 3580–3450 cal yr BP); the middle core at 3140±30 ^14^C yr BP (605 cm depth, intercept: 3370 cal yr BP; 2σ calibration: 3400–3340 cal yr BP). The top at 820±30 ^14^C yr BP (360 cm depth, intercept: 730 cal yr BP; 2σ calibration: 790–680 cal yr BP). Terrestrial plant remains are scarce in the core, limiting an extended chronology.

**Table 1:** Details of the ^14^C age determinations for the core B_22_.

			Calibrated dates BP		Calibrated dates AD/BC		Intercepts	
Code	Depth (cm)	^14^C yr BP	1σ–68%	2σ–95%	1σ–68%	2σ–95%	BP	AD/BC
**Beta-345833**	340	820±30	740–690	790–680	1210–1260	1160–1270	730	1220
**Beta-435399**	605	3140±30	3380–3360	3400–3340	1430–1410	1450–1380	3370	1420
**Beta-345834**	820	3290±30	3560–3470	3580–3450	1610–1520	1630–1500	3540	1590

Cyprus B22 core – Larnaca Salt Lake – Hala Sultan Tekke.

### Pollen and Pollen-slide Charcoal Analyses

A total of 84 samples were prepared for pollen analysis using the standard palynological procedure for clay samples. Pollen grains were counted under x400 and x1000 magnification using an Olympus microscope. Pollen frequencies (%) are based on the terrestrial pollen sum excluding local hygrophytes and spores of non-vascular cryptogams. Aquatic taxa frequencies are calculated by adding the local hygrophytes-hydrophytes to the terrestrial pollen sum. Dinoflagellate cysts were counted on pollen-slides, and are presented as concentrations (cysts per cm^−3^). The fire history was retrieved by counting the pollen-slide charcoal particles (50–200 µm) and expressed as concentrations (fragments per cm^−3^). Concentrations have been plotted on a linear depth-scale. The upper part of the core (250–0 cm depth) is sterile in bioindicators (salty deposits) [50]–[51].

### Numerical Analyses

**Figure 2:**
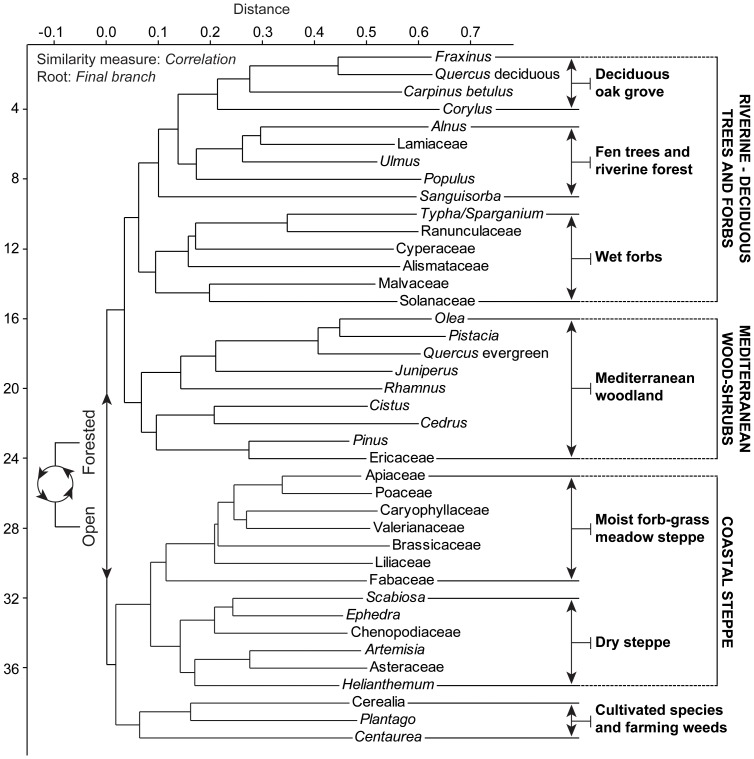
Neighbour Joining analysis of the main taxa from the core B_22_ computed with *correlation* as similarity measure and *final branch* as root. The pollen-types from each cluster were summed to create pollen-derived vegetation patterns.

**Figure 3:**
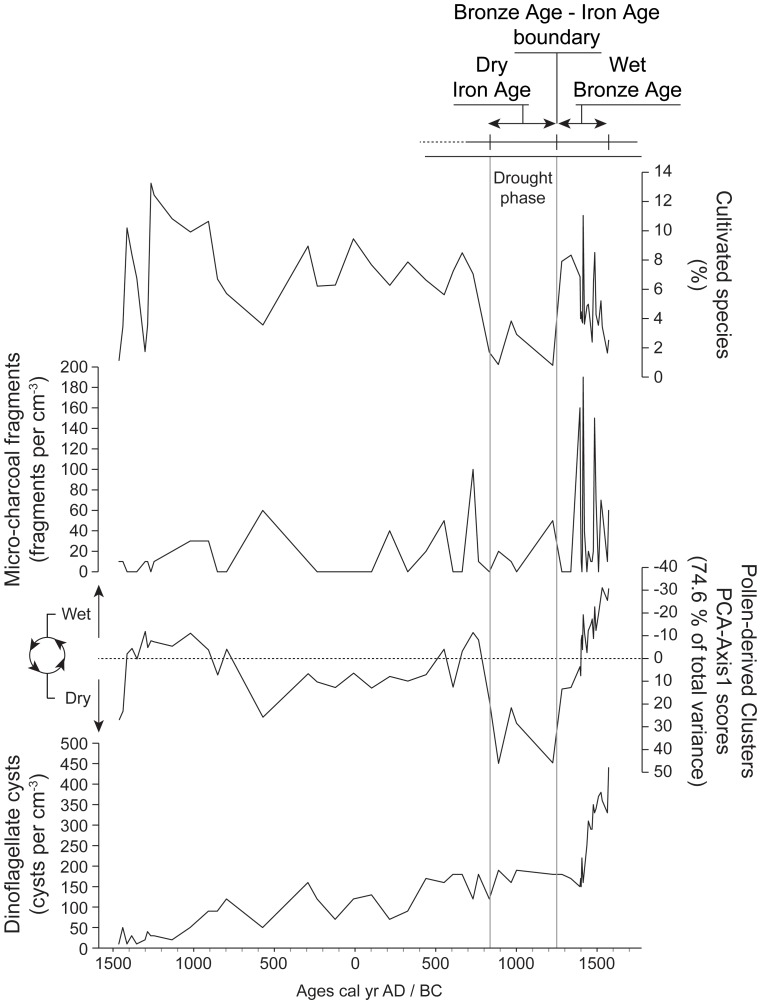
Pollen-derived climatology for the period 3500 years BP to present. The pollen-derived proxy of moisture availability is drawn as PCA-Axis 1 scores (PdVs). The cultivated species, charcoal fragments and dinoflagellate cysts are shown on a linear age-scale. The main climatic event, and the historical-cultural periods are indicated at the top of the diagram.

**Figure 4:**
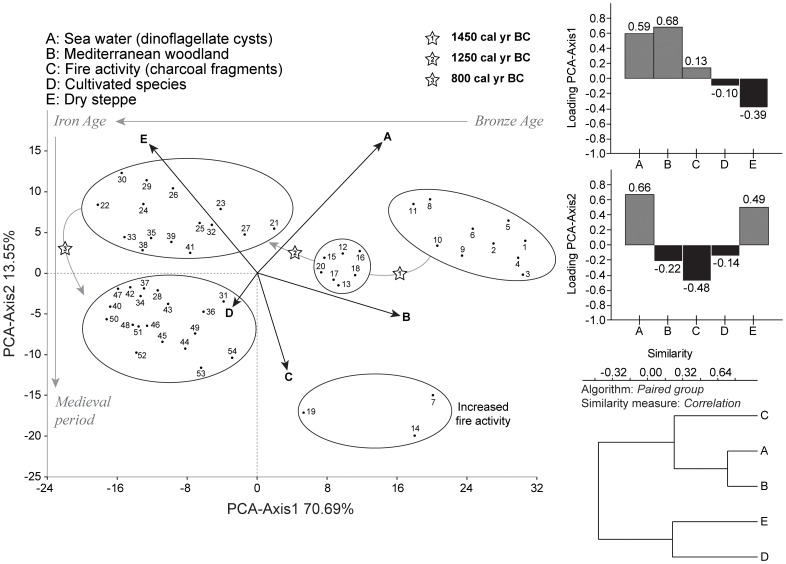
Biplot PCA showing the distribution of samples according to the two main PdVs and environmental factors. PCA axes 1 and 2 account for 84.2% of the cumulative variance. The cluster analysis of the two main PdVs and environmental factors from the core B_22_ was computed with paired group as algorithm, and correlation as similarity measure.

Pollen data were analysed using neighbour joining (NJ) analysis, principal components analysis (PCA), cluster analysis, and linear detrended cross-correlations (_LD_CC). The NJ method is an alternative process for hierarchical cluster analysis, finding hierarchical groupings in multivariate data sets. Here, it is based on pollen-type time-series (presence/absence and abundance). NJ analysis was used to compute the lengths of branches of a tree, using branches as ecological distances between groups of taxa (descending type) [52]. NJ was computed (Fig. 2) using *correlation* as similarity measure and *final branch* as root. The pollen-types from each cluster were summed to create pollen-derived vegetation patterns (PdVs). A PCA was then performed to test the ordination of samples by assessing major changes in PdV-frequencies. The main variance is loaded by the PCA-Axis1. PCA-Axis1 scores have been plotted on a linear depth-scale (Fig. 3). A biplot PCA was finally performed to test the distribution of samples according to the main PdVs and environmental factors (Fig. 4). The ordination of the PdVs was further tested using basic cluster analysis on samples (Fig. 4).

To disentangle if climate proxies from Cyprus and Syria are chronologically linked, _LD_CC (*P = 0.05*) were computed (Fig. 3). The _LD_CC concerns the time alignment of two time series by means of the correlation coefficient (CC). The PCA-Axes1 time-series have been cross-correlated to ascertain the maximal match in time and the potential delay between the two time-series. The CC is then plotted as a function of alignment position. This numerical approach is well-adapted to detect and quantify potential links between environmental data. Positive correlation coefficients are considered, focusing on the Lag_0_ value (with +.50 as significant threshold). Negative correlations are also assessed to test the inverse- or non-correlation between the two time-series (with −.50 as a significant threshold). Non-significant values indicate a complete lack of correlation.

## Results and Discussion

The Larnaca Salt Lake Complex, one of the most important natural standing water bodies in Cyprus, consists of four main lakes [53]. This wetland area, a distinctive coastal lagoon system that has many characteristics of semi-arid temporary salt lakes, is one of the driest parts of the island (precipitation: 351.5 mm per year, mean annual temperatures: 19.6°C), which has been shaped through time by the combined action of temperature, net evaporation and precipitation [54]. A palynological study of modern vegetation in the salt lake from the Akrotiri Peninsula, Cyprus, provides a framework to understand pollen-based reconstructions of ecosystems in this type of environment [55].

### The End of the Marine Environment

Morhange et al. [56] showed that at Kition-Bamboula harbour, the sheltered marine environment turned to a leaky lagoon at ca. 150 cal yr BC, and to a salt lake at ca. 350 cal yr AD. At Hala Sultan Tekke, the presence of *Posidonia oceanica* fibers and rhizomes (Fig. 1) with peaks of dinoflagellate cysts (Fig. 3) in the lower strata indicates a direct marine influence on the deposits [57], and a sheltered marine embayment. The lake complex remained constantly connected to the sea throughout the period ca. 1600–1350 cal yr BC. During this phase, Cypriot ports like Hala Sultan Tekke, Enkomi, and Toumba tou Skourou ensured the availability of various domestic products, such as pottery or copper ingots, and of imported goods such as ivory, tin ingots, Aegean and Levantine pottery [58]. Mass-produced Cypriot pottery was also exported to the Levant [59]. Similar material culture between the urban centers of the northern Levant and Cyprus show close cultural contacts and similar economic functions within the Eastern Mediterranean [58]. The fall of dinoflagellate cysts and *Posidonia* fibers in the sedimentary sequence indicate a shift from sheltered marine to lagoonal environments between ca. 1450 and 1350 cal yr BC. This first environmental shift is concomitant with the decreasing prosperity of the Hala Sultan Tekke harbour. Iacovou [60] mentioned that the settlement of Kition-Bamboula was not nucleated into an urban polity long before the 13^th^ century BC. Kition acquired importance as a port of export for the southern coast only after the harbour of Hala Sultan Tekke had begun to malfunction. The Hala Sultan Tekke harbour, which had served as the port of entry for elite goods from the beginning of the Late Cypriot period (ca. 1600 BC) [61]–[62], started to become the Larnaca Salt Lake (the embayment gradually evolved into lagoon, coastal marsh and finally into an enclosed salt lake), while Kition maintained a commercial port until the Roman period [42], [61].

The changing LBA coastal environment is well-reflected in the core B_22_ pollen record. The two main PdVs (Fig. 2) isolated for the last 3550 years, dry steppe (DS) and Mediterranean woodland (MW), correspond to two contrasted environments (Fig. 3). The important decrease of MW since 1450 cal yr BC may be primarily due to ecological imbalances following the closure of the sheltered marine embayment. This closedown led to the decline of the florishing harbour economy as suggested by Iacovou [60]. The decrease of MW was also possibly related to the important fire activity in the area, probably for agricultural purposes (Fig. 3). The PCA-biplot (Fig. 4) shows that the transition from a MW-dominated to a DS-dominated environment is gradual. The first step was recorded at 1450–1350 cal yr BC, and a second step was reached at ca. 1200 cal yr BC. The drivers of environmental changes for the second step are quite different as no fire activity or changes in the lagoon are attested. The agricultural activity, rich around the site, also strongly declined since 1200 cal yr BC. The PCA-biplot (Fig. 4) indicates that agriculture only became one of the main components of environmental dynamics since ca. 850–750 cal yr BC.

### The Late Bronze Age Crisis

At the end of the 13^th^ century BC, the LBA crisis had affected most of the Eastern Mediterranean and adjacent regions [58], [63]–[65]. By 1100 BC, the settlement patterns and political organization that typified the LBA had come to an end. This major economical breakdown was later offset by a burst of renewed activity [66]–[67] that had repercussions far beyond the original Eastern Mediterranean interaction sphere. New social and economic structures dictated the establishment of new population and power centers during Iron Age (IA) in Cyprus.

Interestingly, major environmental changes occurred at Hala Sultan Tekke during the period encompassing the LBA crisis and the IA I. The dry-saline DS and the wetter MW PdVs correspond to the main loadings in the PCA, explaining most of the variance for the PCA-Axis1 ordination of the data, which accounts for +0.746 of total inertia (Fig. 3). DS (+0.82) is loaded in positive scores, whereas negative values correspond to MW (+0.56) and wet forbs (+0.11). The other clusters show no significant scores (lower than −0.03). DS and MW also constitute two distant branches in the NJ (Fig. 2), and samples during the LBA period are clearly split by these two components (Fig. 4). As the first axis shows a dry *versus* wet time series, variations in PCA-Axis 1 scores may only reflect changes in water availability through the LBA and IA periods.

**Figure 5:**
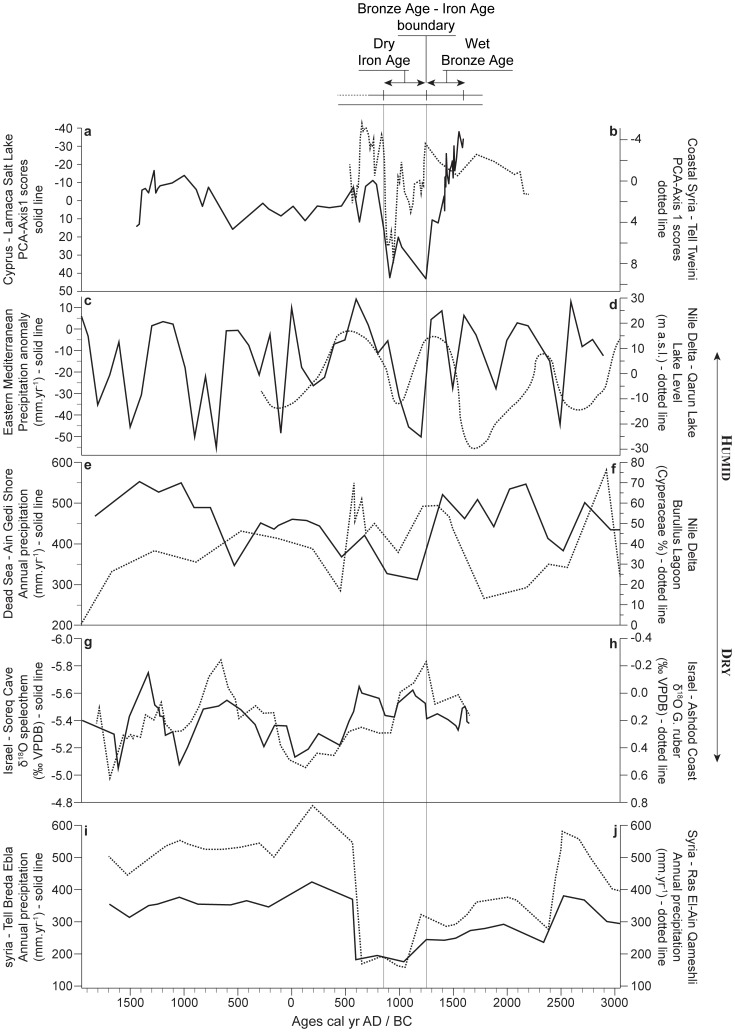
Proxy-based paleoclimate series for the last 5000 years. The vertical stripes represent the timing of the 3200 cal yr BP drought event. (**a**) Pollen-derived climatic proxy from Hala Sultan Tekke, Cyprus, expressed as PCA-Axis1. Negative scores correspond to higher humidity whereas positive scores indicate dry periods; (**b**) Pollen-derived climatic proxy from Tell Tweini, Syria, expressed as PCA-Axis1. Positive scores correspond to higher humidity whereas negative scores indicate dry periods [11]; (**c**) Time-series (5000 BP to present by step of 100 years) of annual precipitation (anomalies from the present in mm/yr) averaged on Southeastern Mediterranean region (longitude 20°E−50°E, latitude 30°N-45°N); the data are reconstructed from pollen data extracted from the European Pollen Database; (**d**) fluctuations in Nile floods, indicated by lake level variations of Lake Qarun (Fayum depression, Egypt) inferred from paleoshorelines and lake sediments [73]; (**e**) reconstruction of annual precipitation based on botanical-climatological transfer functions applied to pollen data from Ain Gedi sediments (Dead Sea shore) [15]; (**f**) Abundance of Cyperaceae pollen, a marker of fresh water input, in a sediment core from Burullus Lagoon in the north-central Nile Delta [14]. Interval of high (low) Cyperaceae pollen percentages are interpreted in terms of increased (decreased) Nile flow; (**g**) fluctuations of the δ^18^O speleothem scores from Soreq Cave, Israel, for the last 3600 years [70]. A lower ratio indicates an increase in humidity; (**h**) fluctuations of the δ^18^O *G. ruber* scores from the core GA-112, Ashdod Coast, Israel [68]–[69]. A lower ratio indicates an increase in humidity; (**i**) Estimated rainfall regimes at Tell Breda, Syria, obtained by correcting mean annual precipitation at Qameshli [71]; (**j**) Estimated rainfall regimes at Ras El-Ain, Syria, obtained by correcting mean annual precipitation at Qameshli [71].

The most positive scores in the PCA-Axis1 are recorded between ca. 1200 and 850 cal yr BC (Fig. 3). The area surrounding Hala Sultan Tekke turned into a drier landscape, the precipitation and groundwater probably became insufficient to maintain sustainable agriculture in this place (Fig. 3). The lower amounts of precipitation at Hala Sultan Tekke, leading to a dry LBA-IA boundary (Fig. 5), are also indicated in the Eastern Mediterranean by marked increases in δ^18^O values on Ashdod Coast [68]–[69] and Soreq Cave [70]. Decreased precipitation at Tell Breda and Ras El-Ain [71], in the Dead Sea [15], as well as reduced Nile floods [14], [72]–[73], minima in the Tigris and Euphrates river discharges [30]–[31], [74], and a dry event in coastal Syria [11] corroborate the hydrologic instability and the extended drought attested in Cyprus during the Late Bronze Age crisis. A vegetation model-based climate reconstruction for the Eastern Mediterranean, reconstructed from pollen data extracted from the European Pollen Database, also indicates a major precipitation anomaly for this period (Fig. 5).

The core B_22_ is consistent with a termination of the drought event during the 9^th^ century BC. In Cyprus, the belated reappearance of state-level polities on the island during the 8^th^ century BC seems incontestable on archaeological grounds [58]. Archaeological data from coastal Syria show that dense occupation reappears at the end of the 9^th^ or the 8^th^ century BC [75]. Egyptian, Aegean, and Assyrian empires also recovered with diversified agro-production, pastoral activities, and sustained a cultural revival during this period [20].

### The Sea Peoples

**Figure 6:**
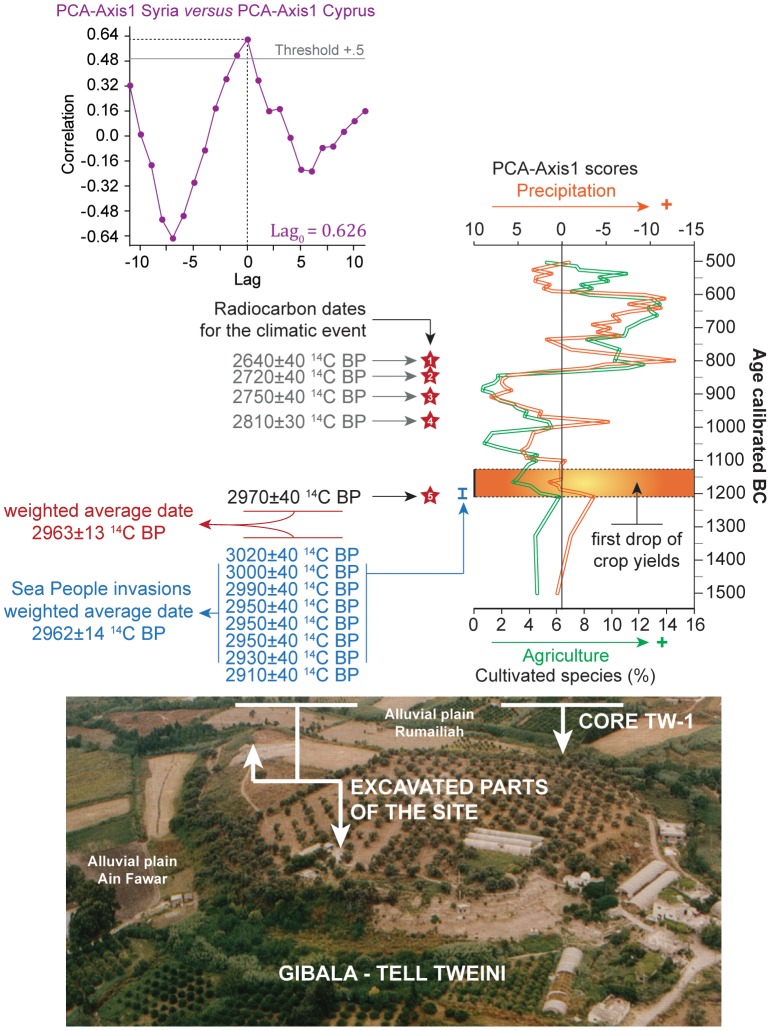
Radiocarbon-based archaeology, climate and agricultural productivity from Gibala-Tell Tweini, Northwest Syria. The pollen-derived climatic and agricultural proxies are plotted against time (1500–500 cal. BC). Radiocarbon dates for the ca. 300 year dry event are indicated with red stars. The time-window of the invasions at the Bronze-Iron Age boundary is framed by radiocarbon dates from the destruction layer (blue). At the top, the cross-correlogram shows the correlation between the pollen-derived proxy of moisture availability from Hala Sultan Tekke and that from Gibala-Tell Tweini. Vertical axes show correlation coefficients while horizontal axes show the lag (1 unit = 1 sample). Significance level *P = 0.05*.

Cyprus offers a valuable case study to look at an ecological-climate influence on past societal shifts. The climate proxies from Hala Sultan Tekke (Fig. 3) and Gibala-Tell Tweini [11]–[12] firmly link the island and the mainland through their comparable climatic evolution. These two time series (PCA-Axes 1), tested by _LD_CC, are significantly correlated with the highest correlation coefficient centered on Lag_0_ (+0.626, *P = 0.05*; Fig. 6). Both proxies reveal a hydrological anomaly for the 1200–850 cal yr BC period, indicating a similar, although not uniform, drought event, recorded both on the island and on the continent. The onset of the drought event seems to be chronologically close to the LBA crisis and the Sea People event.

The LBA crisis, associated with a wave of destructions led by a flow of migrants, the Sea Peoples, is clearly attested in Cyprus with the end of the Late Cypriot IIC period, and the Late Cypriot IIC-IIIA transition dated to 1220–1190 cal yr BC [76]–[77]. Late Cypriot IIC ceramics, imported from Cyprus, were also found at Gibala-Tell Tweini (Fig. 6), in the destruction layer dated from the end of the LBA. Gibala-Tell Tweini was a thriving trade center located on the coast of the ancient Ugarit kingdom, where large-scale excavations over the past 13 years have unearthed a well-preserved destruction layer coeval with the end of the Bronze Age world. A stratified radiocarbon-based archaeology [22] has given the first chronology for the Sea People raids with a pooled mean radiocarbon age of 2962±14 ^14^C yr BP, calibrated to 1 σ at 1215–1190 cal yr BC. This ^14^C calibration range is identical to the Late Cypriot IIC-IIIA transition in Cyprus, indicating a firm chronological link between these events. At the same time, evidence of a long-term dry period, agricultural decline, and subsequent food shortages after 2970±40 ^14^C yr BP [11]–[12] derive from a pollen record from the fertile plains at the bottom of Gibala-Tell Tweini (Fig. 6). The _LD_CC has revealed that this long-term dry period marked by agricultural decline was correlated with the one identified in Cyprus, at Hala Sultan Tekke (Fig. 3). The congruence of drought-induced socioeconomic crisis in the Eastern Mediterranean is confirmed by textual evidence [12], but the human-environmental relationships have remained elusive.

As both archaeological and climatic events (in Cyprus and Syria) were dated by radiocarbon chronology, we choose an objective mathematical approach to propose an unequivocal fit. The first date for the climate event and the fall of crop yields in Syria (core TW1–2970±40 ^14^C yr BP) has been integrated in a matrix of radiocarbon data obtained from the destruction debris (Fig. 6). The numerical analyses show that all the samples pooled in the matrix are statistically the same at the 95% confidence level using a Chi-square (χ^2^) test (sample key 8, where *T* = 6.125<15.5). The weighted average date (2963±13 ^14^C yr BP) gives a 1 σ calibrated age range of 1215–1190 cal yr BC with 33.3% relative probability and another age range of 1180–1160 cal yr BC with 24.3% relative probability, using Calib-Rev. 6.0.1 [49] and Oxcal 4.1 [78] with IntCal09. The weighted average date previously obtained for the first matrix (destruction debris alone) gives a pooled mean age of 2962±14 ^14^C yr BP, with a 1 σ calibrated age range of 1215–1190 cal yr BC with 34.3% relative probability and another age range of 1180–1160 cal yr BC with 26% relative probability. The numerical comparison of 2963±13 ^14^C yr BP and 2962±14 ^14^C yr BP indicates that the two pooled mean ages are statistically the same at the 95% confidence (χ^2^ test, sample key 1, where *T* = 0.002<3.84). The weighted average date obtained by combining these two pooled mean ages is 2962±9 ^14^C yr BP with no changes in the calibrations. This indicates that the LBA crisis, the Sea People raids, and the onset of the drought period are the same event.

Because climatic proxies from Cyprus and coastal Syria are numerically correlated, as the LBA crisis shows an identical calibration range in the island and the mainland, and because this narrative was confirmed by written evidence (correspondences, cuneiform tablets), we can say that the LBA crisis was a complex but single event where political struggle, socioeconomic decline, climatically-induced food-shortage, famines and flows of migrants definitely intermingled.

## Conclusions

By combining data from coastal Cyprus and coastal Syria, this study shows that the LBA crisis coincided with the onset of a ca. 300-year drought event 3200 years ago. This climate shift caused crop failures, dearth and famine, which precipitated or hastened socio-economic crises and forced regional human migrations at the end of the LBA in the Eastern Mediterranean and southwest Asia. The integration of environmental and archaeological data along the Cypriot and Syrian coasts offers a first comprehensive insight into how and why things may have happened during this chaotic period. The 3.2 ka BP event underlines the agro-productive sensitivity of ancient Mediterranean societies to climate and demystifies the crisis at the Late Bronze Age-Iron Age transition.
